# Expanding the landscape of tRNA pathogenic variants in mitochondrial DNA

**DOI:** 10.1093/braincomms/fcag178

**Published:** 2026-05-20

**Authors:** Salvatore Loris Siragusa, Santino Blando, Claudio Fiorini, Alberto Pietro Pasti, Danara Ormanbekova, Francesco Casadei, Luca Morandi, Elena Pegoraro, Rocco Liguori, Valerio Carelli, Chiara La Morgia, Leonardo Caporali, Maria Lucia Valentino

**Affiliations:** Department of Biomedical and Neuromotor Sciences (DIBINEM), Neurology Unit, University of Bologna, 40139 Bologna, Italy; Department of Biomedical and Neuromotor Sciences (DIBINEM), Cellular Signaling Laboratory - Anatomy Center, University of Bologna, 40126 Bologna, Italy; Programma di Neurogenetica, IRCCS Istituto Delle Scienze Neurologiche di Bologna, 40139 Bologna, Italy; Programma di Neurogenetica, IRCCS Istituto Delle Scienze Neurologiche di Bologna, 40139 Bologna, Italy; Programma di Neurogenetica, IRCCS Istituto Delle Scienze Neurologiche di Bologna, 40139 Bologna, Italy; Programma Neuroimmagini Funzionali e MolecolariProgramma Neuroimmagini Funzionali e Molecolari, IRCCS Istituto delle Scienze Neurologiche di Bologna, 40139 Bologna, Italy; Department of Biomedical and Neuromotor Sciences (DIBINEM), Neurology Unit, University of Bologna, 40139 Bologna, Italy; Department of Neuroscience, ERN Neuromuscular Center, University of Padova, 35128 Padua, Italy; Data Science e Bioinformatics Laboratory, IRCCS Istituto delle Scienze Neurologiche di Bologna, 40139 Bologna, Italy; Department of Biomedical and Neuromotor Sciences (DIBINEM), Neurology Unit, University of Bologna, 40139 Bologna, Italy; UOC Clinica Neurologica, IRCCS Istituto delle Scienze Neurologiche di Bologna, 40139 Bologna, Italy; Department of Biomedical and Neuromotor Sciences (DIBINEM), Neurology Unit, University of Bologna, 40139 Bologna, Italy; Programma di Neurogenetica, IRCCS Istituto Delle Scienze Neurologiche di Bologna, 40139 Bologna, Italy; Department of Biomedical and Neuromotor Sciences (DIBINEM), Neurology Unit, University of Bologna, 40139 Bologna, Italy; Programma di Neurogenetica, IRCCS Istituto Delle Scienze Neurologiche di Bologna, 40139 Bologna, Italy; Programma di Neurogenetica, IRCCS Istituto Delle Scienze Neurologiche di Bologna, 40139 Bologna, Italy; Department of Biomedical and Neuromotor Sciences (DIBINEM), Neurology Unit, University of Bologna, 40139 Bologna, Italy; Programma di Neurogenetica, IRCCS Istituto Delle Scienze Neurologiche di Bologna, 40139 Bologna, Italy

**Keywords:** mitochondrial myopathy, mitochondrial DNA, mt-tRNA variant, single muscle fibre microdissection, mtDNA recombination

## Abstract

We present a comprehensive molecular and histopathological characterization of nine patients with mitochondrial myopathy, predominantly manifesting progressive external ophthalmoplegia (PEO), associated with heteroplasmic variants in mitochondrial tRNA genes (mt-tRNA). Among the ten variants identified, four were novel and previously unreported in MITOMAP. Using laser capture microdissection and deep next-generation sequencing, we quantified heteroplasmy at the single-muscle-fibre level, demonstrating that cytochrome c oxidase (COX)–deficient fibres consistently reached near-homoplasmic mutant loads, whereas COX-positive fibres remained heteroplasmic with lower variant fractions. These findings firmly support the pathogenic role of all variants. Furthermore, digital droplet PCR revealed an increased mitochondrial DNA (mtDNA) content in COX-deficient fibres, indicating compensatory mitochondrial biogenesis. Of particular note, one patient harboured two novel heteroplasmic variants, m.10009G > A and m.15961G > A, for which long-read sequencing identified mitogenomes carrying both variants also *in cis*, suggesting the occurrence of mtDNA recombination in human tissue. By applying refined American College of Medical Genetics and Genomics (ACMG) criteria specific for mt-tRNA, we reclassified several variants as pathogenic or likely pathogenic, including three previously deemed of uncertain significance. Overall, our integrative approach—combining single-fibre molecular dissection, mtDNA quantification, and long-read sequencing—broadens the mutational spectrum of pathogenic mt-tRNA variants, highlights the diagnostic value of single-fibre analyses in confirming pathogenicity, and provides new insights into mitochondrial genome dynamics and compensatory responses in mitochondrial disease.

## Teaser

Siragusa *et al*. describe four previously unreported and six rarely reported mitochondrial tRNA variants in nine patients with mitochondrial myopathy, adding evidence for their pathogenic role using single muscle fibres analysis. The study highlights the value of single-fibre analysis in a diagnostic context and for characterization of mitochondrial compensatory mechanisms.

## Graphical Abstract

**Graphical Abstract:**
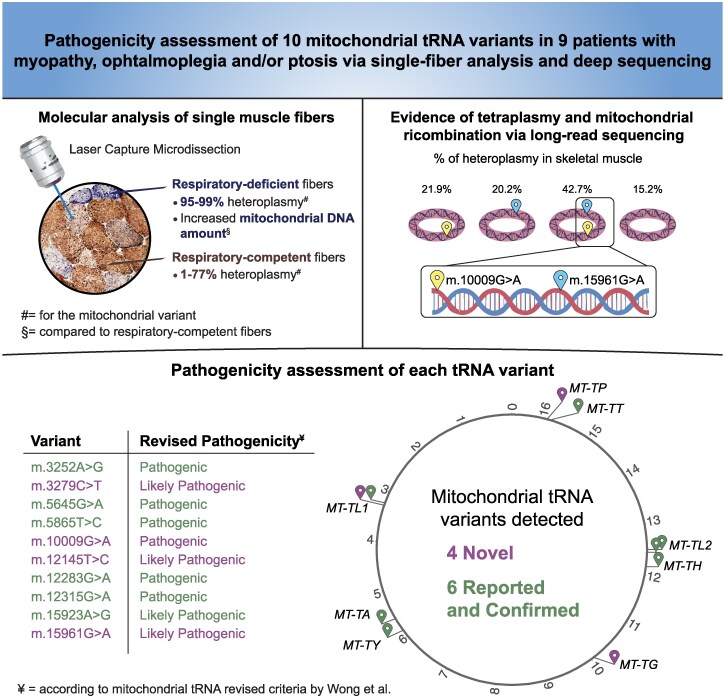

## Introduction

The seminal descriptions of mitochondrial DNA (mtDNA) variants linked to mitochondrial disorder date back to 1988, with the first report of mtDNA macrodeletions in patients with mitochondrial myopathy and progressive external ophthalmoplegia (PEO)^1^ followed by the first missense point mutation associated with Leber hereditary optic neuropathy (LHON).^2^ The following years point mutations affecting mt-tRNA genes were linked respectively to Myoclonic Epilepsy with Ragged Red Fibre (RRF) syndrome (MERRF)^3^ with the m.8344A > G variant in the tRNA^Lys^ gene,^4^ and to Mitochondrial Encephalomyopathy, Lactic Acidosis, and Stroke-like syndrome (MELAS)^5^ with the m.3243G > A variant in the tRNA^Leu^ gene.^6^ These two pathogenic variants remain nowadays the most frequent in MERRF and MELAS.^7^

Currently, 394 variants have been reported associated to diseases across the 22 mitochondrial tRNA (mt-tRNA) genes, as listed in the MITOMAP database^8^ (MITOMAP. https://www.mitomap.org/MITOMAP Accessed 17 November 2025). Only 47 variants are classified as ‘confirmed’ pathogenic or likely pathogenic confirmed variants, while the remaining 347 variants are classified as ‘reported’ or ‘VUS’ or ‘unclear’ or even ‘benign’. Even though all these uncertain variants were associated with several clinical phenotypes, proper functional validation still lacks.

Pathogenic variants in mt-tRNA genes frequently associate with peculiar molecular and clinical hallmarks. For instance, they are typically heteroplasmic, contrary to what happens with the common LHON variants that are usually homoplasmic in affected individuals, as the mt-tRNA pathogenic variant in homoplasmic state may be incompatible with life.^9^ The heteroplasmy implies a segregation of different mutational loads in different tissues or cells of the same tissue, often leading to a widespread clinical spectrum, from very mild to extremely severe phenotypes, and with multisystemic presentation.^9^ However, PEO is a common leading phenotype due to the prevalent muscle involvement, with mitochondrial myopathy histologically hallmarked by a mosaic-like occurrence of cytochrome-c-oxidase (COX or complex IV) negative fibres (lacking completely the enzymatic activity) and/or ragged-red fibres (RRF).^7^ The mosaic composition of skeletal muscle myofibres reflects the variation of heteroplasmic mutant loads in different fibres and even in the same domain of the same myofibre.^10^ This, in turn, is closely related to the heteroplasmic threshold above which the biochemical phenotype—characterized by COX-negative fibres and RRFs—emerges, ultimately determining the clinical manifestations.^9^ This threshold may differ with different mt-tRNA pathogenic variants, and only a minority of studies evaluated thoroughly these issues.

Given the greatly improved sensitivity and affordability of mtDNA sequence analysis, the novel mt-tRNA variants in patients with hallmarks of mitochondrial myopathy or systemic disease are still being described.^11,12^ However, these findings frequently remain without solid support for pathogenicity due to lack of deeper investigations or functional studies. For a long time, the gold standard to demonstrate the pathogenic impact of an mtDNA variant has been the use of the transmitochondrial cytoplasmic hybrid (cybrid) model, which implies long and complex series of investigations.^13^ Alternatively, a recognized and solid proof of pathogenicity can be corroborated by the single myofibre molecular analysis, demonstrating that COX negative/RRFs are tightly associated with very high loads of heteroplasmy or even homoplasmy for the candidate pathogenic variant, as opposed to the normal looking myofibres which remain with a heteroplasmic load under threshold.^14^ Single myofibre microdissection was initially set up with rudimentary methods, which however provided proof of principle evidence connecting the heteroplasmic load with enzymatic deficiency at histoenzymatic staining of muscle sections.^15^ In the last decade, the use of laser capture-based microdissection in combination with digital droplet PCR allows to quantify single fibres heteroplasmy, providing statistically sound results.^10^

In this study we describe a case series of nine patients with mt-tRNA variants, six previously reported^16-24^ and four novel, studied by laser microdissection and genetic analysis of myofibres, validating the pathogenicity of the novel variants, and deepening the investigation of bioenergetic threshold and heteroplasmy segregation.

## Materials and methods

### Patient cohort

Nine patients with evidence of mitochondrial myopathy, in most cases featuring sporadic PEO and ptosis, were included in this case series, recruited during the last two decades at the IRCCS Institute of Neurological Sciences of Bologna and the Neurology Unit of University of Padua. Clinical phenotypes for each of the nine reported patients are described in Table 1. Each recruited patient agreed on the investigation signing an informed consent, after approval of the local Ethical Committee (Ce AVEC 2024/23082—MITOMYOMICS). Furthermore, maternal relatives of the probands were also recruited (*n* = 12), whenever possible, to segregate the genetic defect. For the investigation, we collected skeletal muscle biopsies, peripheral blood samples and urine sedimentary cells. Sample size was determined by the availability of patients with these specific rare variants over the study period.

**Table 1:** Clinical characteristics of the patient cohort, including age at onset, presence of ptosis/ophthalmoplegia, muscle weakness, and additional symptoms

Patient (Sex/Age Of Presentation)	Age At Onset	Ptosis/Ophthalmoplegia	Muscular Weakness	Other Symptoms
**A (M/63y)**	60y	Yes/Yes	No	Parkinson's disease; mild deafness
**B (M/43y)**	7y	Yes/Yes	Proximal, at onset, with facial involvement	Respiratory insufficiency; deafness; cortical and subcortical atrophy and cerebellar atrophy at MRI; mild cognitive impairment; RPE dystrophy with moderate visual impairment
**C (F/72y)**	60y	Yes/No	Proximal	No relevant symptoms
**D (M/64y)**	>40y	Yes/Yes	No	Deafness
**E (F/62y)**	18y	Yes/Yes	Proximal	Retinitis pigmentosa (at onset) with severe visual impairment
**F (M/56y)**	56y	No/No	No	Right lateral homonymous hemianopsia; stroke-like episodes at 56y; bilateral hearing loss
**G (F/62y)**	20y	Yes/Yes	Proximal	Exercise intolerance; mild deafness
**H (F/58y)**	25y	Yes/Yes	Proximal > distal, severe with facial and axial involvement	Respiratory insufficiency; severe dysphagia requiring enteral nutrition; mild deafness; mild cognitive impairment; RPE dystrophy with moderate-severe visual impairment; gastrointestinal dysmotility
**I (M/43y)**	33y	Yes/Yes	No	No relevant symptoms

RPE, Retinal Pigment Epithelium.

### Muscle histology and histochemistry

Muscle specimens were snap-frozen in cooled 2-methylbutane and stored in liquid nitrogen for histological and histoenzymatic analysis including hematoxylin and eosin, Gömöri modified trichrome staining, succinate dehydrogenase (SDH) activity, and double cytochrome-c oxidase/succinate dehydrogenase (COX/SDH) staining according to standard protocols.^10^

### Single muscle fibres isolation by Laser capture microdissection

**Figure 1:**
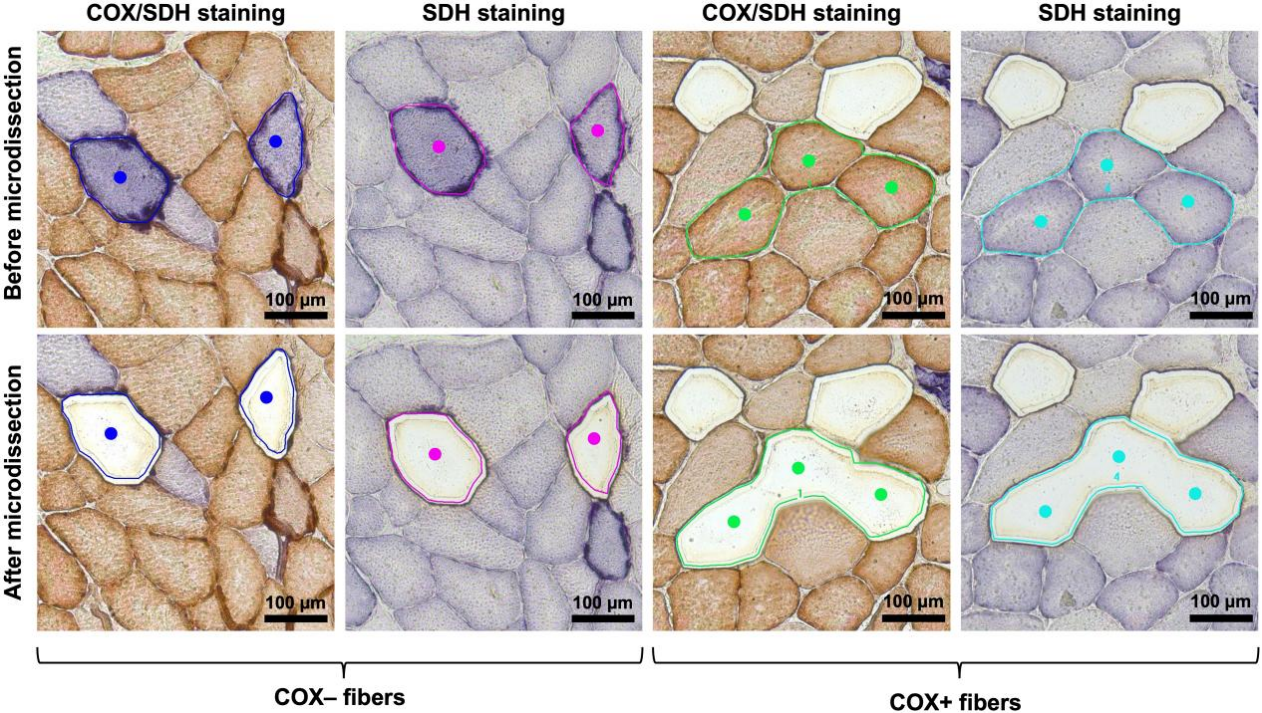
**Laser capture microdissection of COX-deficient and COX-positive skeletal muscle fibres.** Sequential muscle sections were stained for combined cytochrome c oxidase/succinate dehydrogenase (COX/SDH) activity and single SDH activity. In the dual-stained sections, COX-deficient (COX–) fibres appear blue due to the absence of COX activity and preservation of SDH activity, while COX-positive (COX+) fibres appear brownish. The SDH-only staining shows different intensities of blue in colored muscle fibres in relation to the mitochondrial mass. The top panel (Before microdissection) shows targeted fibres highlighted by colored outlines and dots. The bottom panel (After microdissection) demonstrates the precise removal and collection of these specific fibres using a Leica Laser Microdissection system, leaving clear voids in the tissue section.

Snap-frozen liquid nitrogen specimens were sectioned at 10 µm and contiguous sections were placed on 2.0 µm nuclease and human nucleic acid free polyethylene naphthalate (PEN) membrane slides (Leica Microsystems; Milan, Italy). Two slides were prepared, the first with COX/SDH staining, and the second with SDH staining. Based on histochemical staining, 10 COX-negative (COX–) and 10 COX-positive (COX+) muscle fibres from the COX/SDH-stained section were microdissected using Leica LMD 6 Laser Capture Microdissection (LCM) system (Leica Microsystems, Milan, Italy) equipped with a Leica DFC7000 T camera. COX– and COX + microdissected muscle fibres were collected into two different 0.2 mL PCR tubes (Merck KGaA, Darmstadt, Germany), as depicted in Fig. 1. Subsequently, the contiguous section stained with SDH, corresponding to the previously used COX/SDH section, was selected for microdissection and separate collection of the same muscle fibres that had been collected from the COX/SDH-stained section.

Laser captured fibres were incubated with a solution containing 50 mM TRIS HCl pH 8.5; 6.9 μM proteinase K (Cell Signalling Technology, Danvers, MA, USA) and 0.1% TWEEN 20 (Merck KGaA, Darmstadt, Germany) into a thermal cycler for 16 h 55°C and 10 min 95°C to extract total genomic DNA used in target genetic screenings of putative mtDNA pathogenic variants.

### mtDNA short reads sequencing and heteroplasmy analysis

The complete mtDNA sequencing was performed on total DNA derived from skeletal muscle biopsies of the probands, using a standard phenol-chloroform extraction protocol. The Next Generation Sequencing (NGS) libraries were prepared with the xGen™ DNA Library Prep EZ kit and the mtDNA was enriched using the XGen™ Human Mitochondrial DNA Hybridization Panel (Integrated DNA Technologies, Inc., Iowa, USA).

On laser-microdissected muscle fibres from COX/SDH-stained sections, we performed target NGS for accurate detection and heteroplasmy quantification of variants of interest. A PCR amplicon of ∼300–400 bp including the studied variants was obtained from lysed muscle fibres, using the PrimeSTAR Max DNA Polymerase (Takara Bio, Japan), with specific primers and conditions available upon request. The same approach was adopted for blood cells and urinary sediment available samples of probands’ maternal relatives. The purified PCR products were prepared for NGS with the xGen™ DNA Library Prep EZ kit, following manufacturer’s instructions.

All sample libraries were sequenced on a MiSeq System (Illumina, San Diego, CA, USA) with 2 × 150 bp paired-end reads. The fastq files were analyzed using the mtDNA-Server 2 pipeline,^25^ then manually reviewing the output annotated vcf files. The allele fraction detected for each variant was considered as its heteroplasmic fraction. Haplogroup assessment was performed using Haplogrep 3 (https://haplogrep.i-med.ac.at).^26^ For the detection of mtDNA structural rearrangements from NGS data, we deployed the MitoSAlt tool with default configuration settings for enriched mtDNA sequencing.^27,28^

### Quantification of mtDNA copy number and content using ddPCR

Digital droplet PCR (ddPCR) is a quantitative PCR-based technique employed to assess mtDNA copy number (CN) in bulk muscle tissue and to quantify mtDNA density in individual SDH-stained muscle fibres previously isolated by LCM. Due to the known interfering effects of 3,3′-diaminobenzidine (DAB), a chromogenic reagent used in COX histochemistry, on ddPCR proper functioning,^29^ only SDH-stained sections were used for single fibre mtDNA analysis. To avoid potential biases in mtDNA copy number quantification introduced by the loss of nuclear material, we opted to assess mtDNA density based on the area of microdissected muscle fibres. UV-based laser capture microdissection relies on laser-induced photoablation to isolate tissue regions of interest.^30^ However, this approach may result in partial exclusion of peripheral regions where myonuclei are typically located (Fig. 1). Since nuclear DNA is required as a reference for mtDNA copy number calculations, such loss could skew normalization. Therefore, instead of calculating absolute mtDNA copy number, we measured mtDNA amount by normalizing the number of *MT-ND2*–positive droplets to the total area of microdissected fibres, providing a reliable estimate of mtDNA content independent of nuclear inclusion. The mtDNA copy number is based on duplex amplification with specific probes of nuclear and mitochondrial DNA and expressed as a ratio of mtDNA and nDNA multiplied by two. The mtDNA primers used were designed on *MT-ND2* gene: mitochondrial-forward primer (CACAGAAGCTGCCATCAAGTA), mitochondrial-reverse primer (CCGGAGAGTATATTGTTGAAGAG) with probe: (FAM/CCTCACGCAAGCAACCGCATCC/BHQ1). The primers for nuclear gene were designed on *FASLG* gene: nuclear-forward primer (GGCTCTGTGAGGGATATAAAGACA), nuclear-reverse primer (CAAACCACCCGAGCAACTAATCT) with probe: (HEX/CTGTTCCGTTTCCTGCCGGTGC/BHQ1). The reaction was conducted using ddPCR Supermix for Probes (No dUTP) (Bio-Rad; Milan, Italy) in accordance with the manufacturer’s instructions, with DNA properly diluted in molecular-grade water. The analysis was performed on the QX200 ddPCR System (Bio-Rad; Milan, Italy). Data processing and analysis were carried out using Quantasoft Analysis Pro 1.0 software (Bio-Rad; Milan, Italy). To obtain the copy number in bulk samples, we calculated the ratio of *MT-ND2* to *FASLG* and multiplied the result by 2.

### Whole exome sequencing

Whole exome sequencing (WES) sample library was prepared from genomic DNA using xGen DNA Library Prep Kit EZ (IDT) and enriched using xGen Exome Hyb Panel v2 (IDT). The sequencing was performed on a NovaSeq 6000 instrument (Illumina) with 150 bp paired-end reads. Bioinformatic analysis followed the GATK Best Practices workflow for germline variant discovery, aligning to reference genome GRCh38/hg38; called variants were annotated using Ensembl Variant Effect Predictor (VEP).^31^ Rare variants in genes included in the Panel for Congenital myopathy (v6.34) and Mitochondrial DNA maintenance disorder (v3.6) of Genomics England PanelApp (https://panelapp.genomicsengland.co.uk) were analyzed and classified using American College of Medical Genetics and Genomics (ACMG) criteria.^31^

### mtDNA long reads sequencing

Long-read sequencing was performed exclusively on Patient E, who carried two distinct mtDNA variants (m.10009 G > A and m.15961 G > A). The aim was to assess the distribution of both variants across blood, urinary sediment, and skeletal muscle tissue. A 9309 bp region of interest, spanning from nucleotide position m.8896 to m.1636, was amplified to encompass both variants identified in Patient E. The amplified products from each tissue were sequenced using GridION platform (Oxford Nanopore, UK) with the Native Barcoding Kit 24 V14 (SQK- NBD114.24). From long reads aligned bam files using MiniMap2, the reads containing the two variants, singularly or in *cis*, were individually counted to obtain heteroplasmy estimates as evidence of mtDNA recombination.

### Grading pathogenicity of mt-tRNA variants

All mitochondrial variants identified in this study were interpreted according to the Wong *et al.*^16^ refined ACMG guidelines tailored for mt-tRNA variants. This criterium incorporates mitochondrial-specific considerations such as heteroplasmy level, tissue-specific expression, functional assessment, inheritance patterns, and disease mechanisms. Each variant was assessed using curated criteria and classified into standard categories: pathogenic (P), likely pathogenic (LP), uncertain significance (VUS), likely benign (LB), or benign (B). The scoring system for combining criteria to classify sequence variants are the same as those of the ACMG guidelines.^31^ Interpretation was stated starting from our results and supported by publicly available databases including MITOMAP,^8^ as well as primary literature and, when available, functional and clinical data.

### Statistical analysis

Statistical analyses were performed using GraphPad Prism version 10.4.2 (GraphPad Software, San Diego, CA, USA). The relationship between age and mtDNA deletion load was assessed using simple linear regression separately for patients and controls. Linear regression lines were compared using an analysis of covariance (ANCOVA) approach implemented in GraphPad Prism to test for differences in slopes and intercepts (elevations) between groups. When no significant difference in slopes was detected, a pooled slope was calculated using all data points. A two-sided *P* value < 0.05 was considered statistically significant.

## Results

### Patients’ medical histories

The clinical features of the nine patients are summarized in Table 1; each one is codified with a letter (A–I). Except for Patients C and F, all individuals presented PEO and ptosis. Five patients experienced proximal muscular weakness with some facial involvement at disease onset, while four patients did not show any degree of muscular weakness. Notably, in several cases, patients exhibited additional systemic features including respiratory insufficiency, brain and cerebellar atrophy, stroke-like episodes, hearing loss, retinal dystrophy, gastrointestinal symptoms, and cognitive impairment and parkinsonism. Age at onset ranged from childhood to late adulthood (7–60 years).

The clinical description of each patient is provided in Supplementary Materials.

### Muscle histopathology

**Figure 2:**
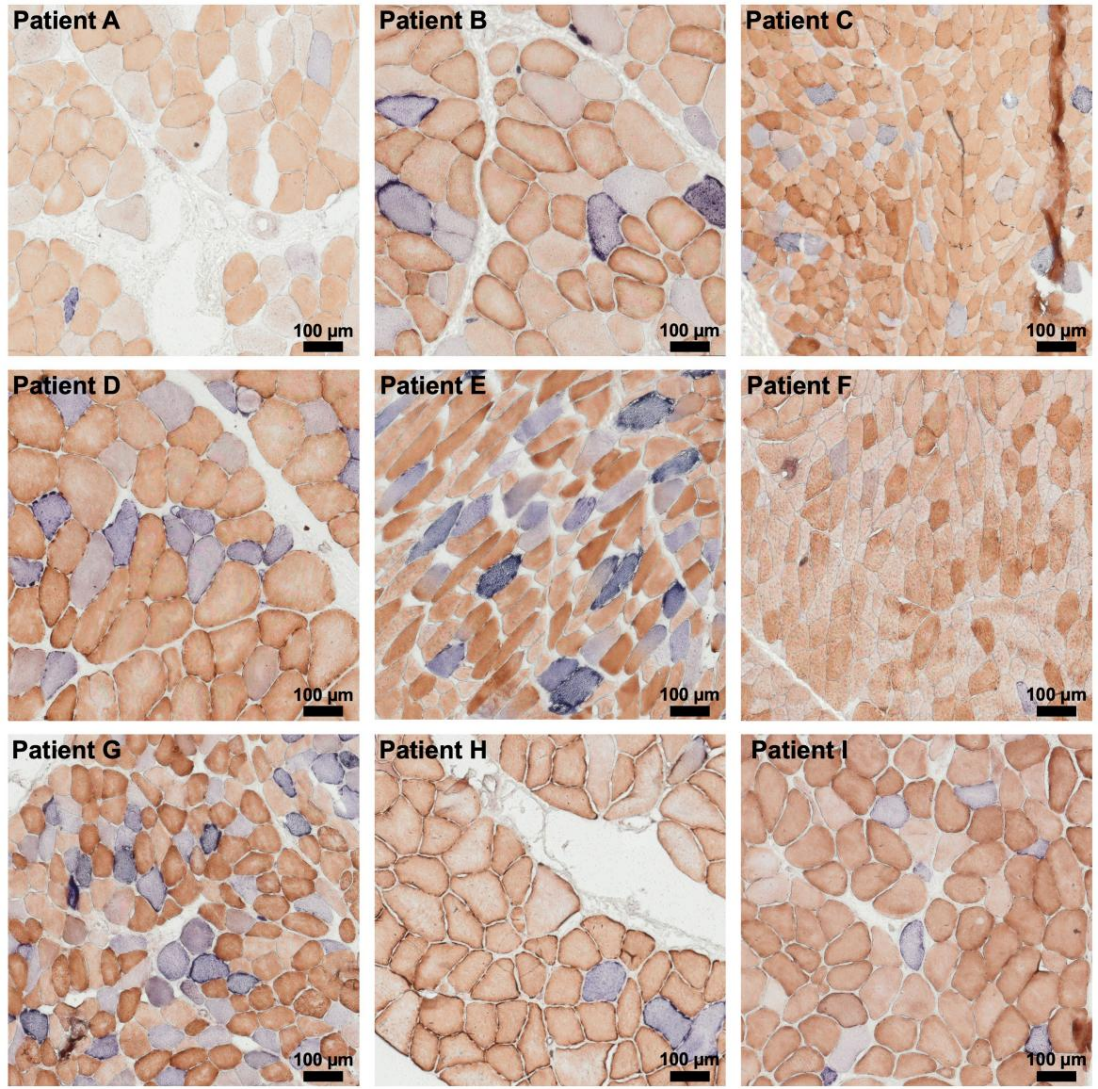
**Representative COX/SDH histoenzymatic staining from skeletal muscle biopsies of all nine patients.** Panels (A-I) show representative COX/SDH histoenzymatic staining of all patients (in order A-I), highlighting mitochondrial respiratory chain dysfunction at the single-fibre level. Fibres lacking COX activity but retaining SDH activity (COX– fibres) therefore appear blue, whereas metabolically intact fibres (COX+ fibres) appear brown. In both staining, the colour intensity reflects the activity of the putative enzyme. The mosaic distribution of COX– and COX+ fibres reflects heteroplasmic segregation of mtDNA variants within skeletal muscle, clearly visible in most samples. COX: cytochrome oxidase c/complex IV; SDH: succinate dehydrogenase/complex II.

The COX/SDH staining was used to evaluate the muscle fibres with deficient mitochondrial oxidative function. COX (complex IV) is a mitochondrial enzyme encoded by both mitochondrial and nuclear DNA, whereas SDH (complex II) is only contributed by nuclear genes. Thus, in COX/SDH staining, if there is normal enzymatic function, the cells have brown staining (COX yellowish + SDH blue) whereas, if defective mtDNA occurs, the cells will maintain only the SDH activity losing the COX staining, displaying a blue colour. Histopathological examination of skeletal muscle biopsies from all nine patients revealed variable degrees of mitochondrial dysfunction. COX/SDH histoenzymatic staining (Fig. 2) consistently showed the presence of COX– blue fibres, with a typical mosaic pattern of COX– and COX+ fibres. The percentage of COX– fibres varied significantly among patients, ranging from as low as 0.1% in Patient F to as high as 26% in Patient G. The COX– fibre percentages for the remaining patients were as follows: A, 1%; B, 10%; C, 5%; D, 14%; E, 18%; H, 2%; and I, 7% (see also Table 2 for quantitative data).

**Table 2:** Genetic evaluation and heteroplasmy analysis of mitochondrial tRNA mutations in bulk tissue and microdissected single muscle fibres

Patient	Variant	Gene	Heteroplasmy			% COX– fibres	% RRFs/total fibres	% mtDNA CN change (control range: –37%/+36%)	% Single COX- fibres mtDNA amount change
			Bulk	COX+ fibres	COX– fibres				
**A**	m.3252A > G	*MT-TL1*	37%	8%	99%	1%	0.1%	+20%	+31%
**B**	m.3279C > T	*MT-TL1*	35%	16%	98%	10%	1.3%	–19%	+295%
**C**	m.5645G > A	*MT-TA*	45%	24%	99%	14%		–17%	–6%
**D**	m.5865T > C	*MT-TY*	43%	41%	95%	5%		+26%	+164%
**E**	m.10009G > A m.15961G > A	*MT-TG* *MT-TP*	61% 70%	56% 60%	98% 99%	18%	1.9%	+43%	+265%
**F**	m.12145T > C	*MT-TH*	76%	77%	99%	0.1%		–30%	+143%
**G**	m.12283G > A	*MT-TL2*	40%	6%	95%	26%	0.2%	–4%	+146%
**H**	m.12315G > A	*MT-TL2*	9%	1%	97%	2%	0.4%	–4%	+6%
**I**	m.15923A > G	*MT-TT*	23%	32%	98%	7%		–20%	+400%

CN, copy number; *MT-TA*, mitochondrial tRNA alanine; *MT-TG*, mitochondrial tRNA glycine; *MT-TH*, mitochondrial tRNA histidine; *MT-TL1*, mitochondrial tRNA leucine 1; *MT-TL2*, mitochondrial tRNA leucine 2; *MT-TP*, mitochondrial tRNA proline; *MT-TT*, mitochondrial tRNA threonine; *MT-TY*, mitochondrial tRNA tyrosine; RRFs, ragged-red fibres.

**Figure 3:**
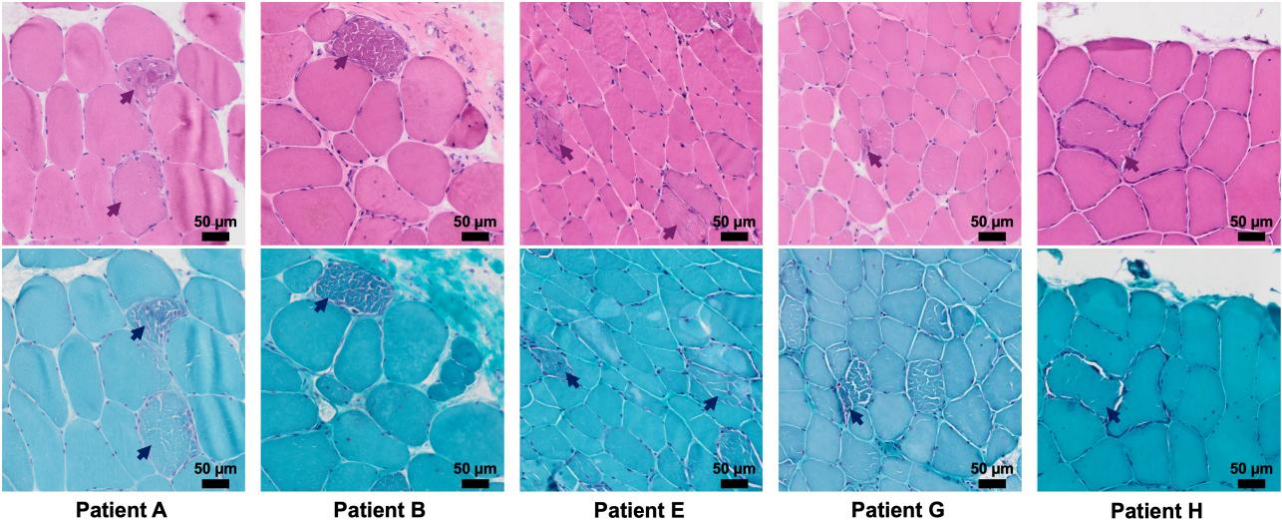
**Representative images of ragged-red fibres (RRFs) from the five patients (A, B, E, G, and H).** The top row shows Hematoxylin and eosin staining, which highlights overall muscle fibre morphology and cytoplasmic basophilia, revealing subsarcolemmal basophilic accumulations in affected fibres (arrows). The bottom row shows modified Gömöri trichrome staining, used to visualize abnormal mitochondrial proliferation; it reveals irregular subsarcolemmal deposits with a fragmented, ‘cracked’ appearance, visible as dark violet to bluish rims. These features are characteristic of RRFs in mitochondrial myopathy.

In addition, five patients (A, B, E, G, and H) exhibited ragged-red fibres (RRFs), which are characterized by subsarcolemmal accumulation of abnormal mitochondria and are considered a hallmark of mitochondrial myopathy.^3,4,14^ These were clearly visualized by both hematoxylin and eosin and modified Gömöri trichrome staining, as depicted in Fig. 3.

These findings document the mitochondrial aetiology of myopathy and underscore the histoenzymatic staining as instrumental in assessing the bioenergetic impact of mtDNA mutations.

### Sequence analysis and mtDNA assessment

**Figure 4:**
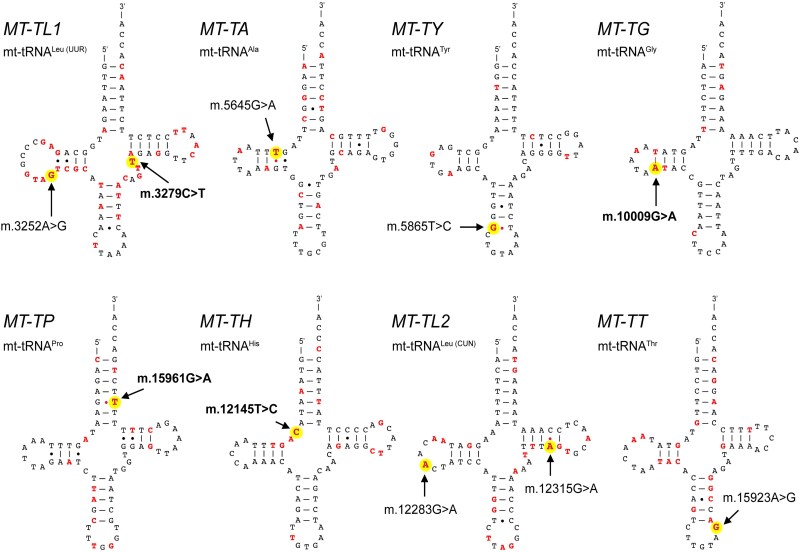
**Structural location of reported, confirmed, and novel mt-tRNA variants.** For each mt-tRNA discussed in this article, currently known variants from MITOMAP (MITOMAP. https://www.mitomap.org/MITOMAP Accessed 17 November 2025) are shown in red, including reported and confirmed ones. Variants analyzed in this study are also shown in red and highlighted with a bright yellow background. Novel variants identified in our cohort are indicated in bold.

Sequence analysis of mtDNA extracted from skeletal muscle identified heteroplasmic variants in mt-tRNA genes across all nine patients (Fig. 4). No pathogenic or likely pathogenic variants were detected by WES in both Congenital myopathy and Mitochondrial DNA maintenance disorder genes panels (data not shown), suggesting that mitochondrial variants were most likely to be associated with the phenotype. At WES analysis, we only detected a heterozygous variant in Patient H in the *SUCLA2* gene classified as VUS. In addition, mtDNA copy number analysis from bulk muscle tissue did not reach any significant difference between patients (mtDNA copy number = 2241 ± 559, mean ± SD, *n* = 9) and controls (mtDNA copy number = 2251 ± 598, mean ± SD, *n* = 5) (Table 2). Furthermore, we assessed the occurrence of mtDNA rearrangements in both controls and patients, excluding the presence of deletions or duplications at total heteroplasmy levels above 1%. However, we found an expected age-dependent mild accumulation of multiple deletions in both patients and controls, with a significantly higher load of deletions in the patients’ cohort as compared to controls (Supplementary Fig. 1). Instead, assessment of mtDNA duplications revealed no significant differences between the two groups, with mostly overlapping profiles (data not shown).

Below, we provide a case-by-case summary of mtDNA findings for each proband, including heteroplasmy levels in different tissues, mtDNA haplogroup background, variant location within the mt-tRNA structure (Fig. 4), and available results from maternal relatives.

#### Patient A

In this male patient we identified the m.3252A > G variant in the *MT-TL1* gene, affecting the DHU loop of the tRNA^Leu(UUR)^ (haplogroup T2c1d1). This variant, present at 37% heteroplasmy in muscle, has been previously reported in association with mitochondrial encephalomyopathy, pigmentary retinopathy, dementia, hypoparathyroidism and diabetes mellitus and is classified as pathogenic in MITOMAP.^17^ The *MT-TL1* gene has numerous other confirmed or reported pathogenic variants as depicted in Fig. 4. Blood samples collected in 2012 and 2018 showed 7% and 6% heteroplasmy, respectively. The variant was not detected in the blood or urinary sediment of four sisters and a maternal nephew.

#### Patient B

This male patient carried the m.3279C > T variant in the *MT-TL1* gene (haplogroup J1c1), located in the variable loop of the tRNA^Leu(UUR)^. The variant, which is novel and not reported in databases, showed 35% heteroplasmy in muscle. Heteroplasmy levels were 11% and 12% in blood cells (respectively in 2016 and 2017) and 76% in urinary sediment in 2017. The mother and brother were also analyzed and, although the variant was not detected in their blood, the urinary sediment showed heteroplasmy of 7% and 2%, respectively.

#### Patient C

In this female patient we detected the m.5645G > A variant in the *MT-TA* gene at 45% heteroplasmy in muscle (haplogroup HV). This variant affects the DHU-stem of the tRNA^Ala^ and was firstly reported by Wong *et al.*^16^ Blood and urinary sediment analysis revealed low heteroplasmy (2% and 6%, respectively), while the sister’s urinary sediment tested negative.

#### Patient D

In this male patient we found the m.5865T > C variant in the *MT-TY* gene, affecting the anticodon stem of the tRNA^Tyr^. Mutant load in skeletal muscle was at 43% heteroplasmy (haplogroup H6a1b2) and this variant was previously described by Wong *et al*.^16^ Blood testing showed low heteroplasmy: 2% in 2007 and 1% in 2016. Segregation analysis of the mtDNA variant was not performed as the patient’s mother was deceased and a sister was not available due to advanced age, but both were reported as not having ptosis.

#### Patient E

This female patient uniquely exhibited two distinct mt-tRNA mutations in skeletal muscle: m.10009G > A in *MT-TG* (61% heteroplasmy) and m.15961G > A in *MT-TP* (70% heteroplasmy), affecting the DHU-stem of tRNA^Gly^ and the acceptor stem of tRNA^Pro^, respectively. Both variants are novel (haplogroup H2c1). In blood, the m.15961G > A variant had 63% heteroplasmic load, while in urine sediment was 69%. The m.10009G > A variant was undetectable in blood but present at 25% heteroplasmic load in urinary sediment. In the proband’s daughter, the m.15961G > A variant was present in both blood (52% heteroplasmy) and urine (45% heteroplasmy), while m.10009G > A was absent. The two variants were not found in the blood or urinary sediment of the proband’s sister.

**Figure 5:**
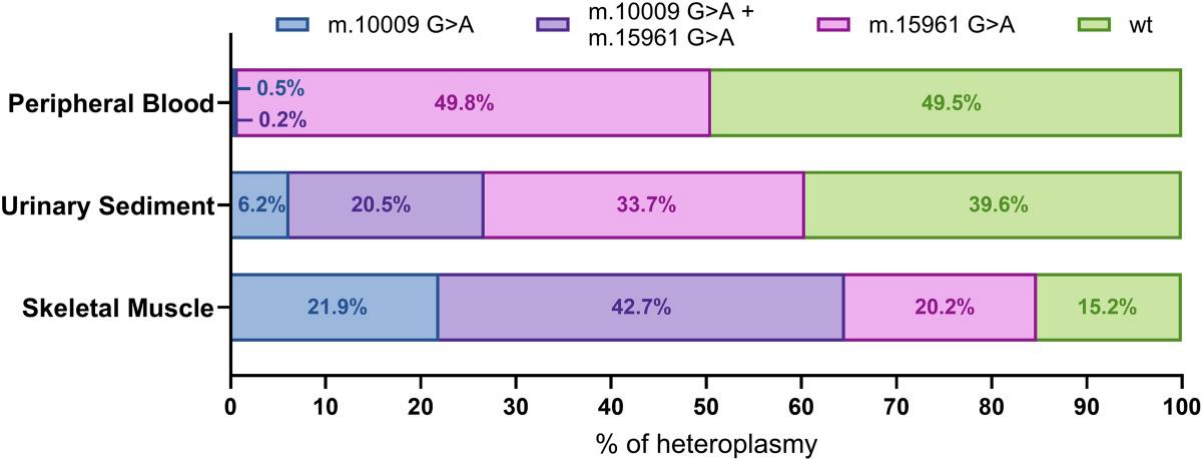
**Percentages of mtDNA molecular species in different tissues of patient E assessed by long read sequencing.** Stacked bar plots show the relative proportions of mtDNA molecular species detected in peripheral blood, urinary sediment, and skeletal muscle using Oxford Nanopore Technology long-read sequencing. Green indicates wild-type (wt) mtDNA; blue indicates mtDNA carrying the m.10009G > A variant alone; pink indicates mtDNA carrying the m.15961G > A variant alone; purple indicates mtDNA molecules harboring both variants in cis (m.10009G > A + m.15961G > A). Percentages represent heteroplasmy levels calculated from aligned long read sequences.

To assess the *cis/trans* occurrence of the two mtDNA variants detected in Patient E on the individual mtDNA molecule, we quantified heteroplasmy levels of m.10009 G > A and m.15961 G > A in peripheral blood, urinary sediment, and skeletal muscle using Oxford Nanopore Technology long-read sequencing (see Materials and Methods). As shown in Fig. 5, in peripheral blood we clearly detected wild-type and m.15961 G > A heteroplasmic variant, whereas either the m.10009 G > A alone or the combination of both variants were essentially absent (0.5% and 0.2% respectively). These values are within the error rate of the method (∼1%),^32^ thus we considered them artefactual. In contrast, urinary sediment and skeletal muscle exhibited all four molecular species, namely the wild type form, each variant individually, and molecules carrying both variants in *cis*—at different levels of heteroplasmy in both tissues (Fig. 5).

#### Patient F

In this male patient we found the m.12145T > C variant in the *MT-TH* gene, located in the DHU-loop of the tRNA^His^, detected at 76% heteroplasmy in skeletal muscle (haplogroup T2b). This mutation is novel and has not been previously reported. Segregation analysis among family members was not possible due to the absence of relatives’ samples, as the patient was lost at follow-up.

#### Patient G

In this female patient we detected the m.12283G > A variant in *MT-TL2*, affecting the DHU-loop of tRNA^Leu(CUN)^, at 40% heteroplasmy in skeletal muscle (haplogroup H3). This was previously reported in a patient with PEO and ophthalmoplegia^18^ and is listed in MITOMAP as a variant of uncertain significance. Minor heteroplasmy levels were found in urine (4%) and blood (1%). Her family history was negative for ptosis and other muscle disorders. Segregation analysis of this variant was not performed due to the advanced age of her family members.

#### Patient H

This female patient harboured a mutation in *MT-TL2*: the m.12315G > A variant in the T-stem of the tRNA^Leu(CUN)^ (haplogroup J1c5d). She showed 9% heteroplasmy in muscle, and this variant is classified as likely pathogenic in MITOMAP. It was initially reported by Fu *et al.*^19^ in a patient with PEO, ptosis, limb weakness, hearing loss, and pigmentary retinopathy. The variant was absent in the blood of both the proband and her brother.

#### Patient I

This male patient displayed the m.15923A > G variant in the *MT-TT* gene, affecting the anticodon loop of the tRNA^Thr^, at 23% heteroplasmy in muscle (haplogroup K1a). This variant has been previously described in two infants with fatal infantile respiratory chain deficiency.^20^ Patient’s mother was not available for the segregation analyses.

### Molecular genetic investigations in single skeletal muscle fibres

Next, to test the pathogenic significance of these variants at the cellular level in the skeletal muscle biopsies, we quantified heteroplasmy in COX– and COX+ fibres as compared to bulk tissue from each patient. As shown in Table 2, COX– fibres consistently displayed near-homoplasmic levels of the mutant allele (>95%), regardless of the variant. By contrast, COX+ fibres exhibited variable and generally lower heteroplasmic fractions, in many cases remaining below the presumed biochemical threshold. Ultimately, heteroplasmy levels from bulk muscle tissue ranged from 9% to 76%.

Additionally, mtDNA amount normalized by area revealed higher mtDNA content in COX– fibres compared with COX+ fibres across most patients, compatible with the compensatory mitochondrial proliferation. Notably, this shift in mtDNA amount was particularly marked in patients B, D, E, G, and I, who also showed a consistent percentage of COX– fibres.

### Pathogenicity grading of mt-tRNA variants

Variant classification was performed following Wong *et al.* refined ACMG for mt-tRNA variants,^16^ integrating MitoTIP scores,^34^ structural localization within the mt-tRNA molecule, and available literature and our studies (see Table 3). Notably, most variants were classified as likely pathogenic or pathogenic, with key supporting criteria such as PS3, PM7, PM8 and PM9.

**Table 3:** Pathogenicity assessment of mitochondrial tRNA variants in nine patients

Patient	Variant	Gene	Mitotip^33^	Location	Reported Status^a^	Revised Criteria	Revised Scores
**A**	m.3252A > G	*MT-TL1*	39.4	DHU-loop	Cfrm[LP]^16,17,21^	PS3, PS5, PM7, PM8, PM9	P (14)
**B**	m.3279C > T	*MT-TL1*	41.7	Variable loop	Novel	PS3, PM7, PM8, PP7	LP (9)
**C**	m.5645G > A	*MT-TA*	69.4	DHU-stem	VUS^16^	PS3, PM7, PM8, PM9	P (10)
**D**	m.5865T > C	*MT-TY*	61.9	Anticodon Stem	VUS^16^	PS3, PM7, PM8, PM9	P (10)
**E**	m.10009G > A m.15961G > A	*MT-TG* *MT-TP*	12.7 29.0	DHU-stem Acceptor Stem	Novel Novel	PS3, PM8, PM9, PP3, PP7 PS3, PM7, PM8	P (10) LP (8)
**F**	m.12145T > C	*MT-TH*	48.6	DHU-loop	Novel	PS3, PM7, PP6, PP7	LP (8)
**G**	m.12283G > A	*MT-TL2*	43.0	DHU-loop	VUS^18,24^	PS3, PM7, PM8, PM9	P (10)
**H**	m.12315G > A	*MT-TL2*	82.2	T-stem	Cfrm[LP]^19,22,23^	PS3, PS5, PM7, PM9	P (12)
**I**	m.15923A > G	*MT-TT*	46.8	Anticodon loop	Cfrm[LP]^20^	PS3, PM8, PM9	LP (8)

Cfrm, Confirmed; LP, Likely Pathogenic; *MT-TA*, mitochondrial tRNA alanine; *MT-TG*, mitochondrial tRNA glycine; *MT-TH*, mitochondrial tRNA histidine; *MT-TL1*, mitochondrial tRNA leucine 1; *MT-TL2*, mitochondrial tRNA leucine 2; *MT-TP*, mitochondrial tRNA proline; *MT-TT*, mitochondrial tRNA threonine; *MT-TY*, mitochondrial tRNA tyrosine; P, Pathogenic; VUS, Variant of Uncertain Significance.

^a^Pathogenicity status was extrapolated from MITOMAP (MITOMAP. https://www.mitomap.org/MITOMAP Accessed 17 November 2025).

In particular, the variants identified in Patients A (m.3252A > G, *MT-TL1*)^16,17,21^ and H (m.12315G > A, *MT-TL2*),^19,22,23^ initially annotated as likely pathogenic (LP), were reclassified as pathogenic (P) due to the incorporation of strong functional evidence criteria, such as PS3, PS5, PM9. Three variants recognized in Patients C (m.5645G > A, *MT-TA*),^16^ D (m.5865T > C, *MT-TY*)^16^ and G (m.12283G > A, MT-TL2)^18,24^ were previously reported as VUS. These variants had been documented in independent families and the PM9 criterion remained applicable. Therefore, in combination with our analyses, these variants were reclassified as pathogenic.

This reclassification was supported by multiple converging findings: high frequency of COX-deficient fibres with near-homoplasmic loads detected via deep NGS on microdissected single fibres, and a consistent increase in mtDNA amount in COX– fibres, reflecting compensatory mitochondrial biogenesis.

## Discussion

Our study provides an in-depth investigation of nine probands, most of them with PEO, associated with mtDNA point mutations affecting mt-tRNA genes. Four of the variants were novel and, combining different metrics including the single muscle fibre assessment of heteroplasmy comparing COX– and COX+ fibres, we classified one as pathogenic and three as likely pathogenic. We also revaluated the pathogenicity score of the remaining six variants, which were already reported^16-24^ with different classifications on MITOMAP. By applying a combination of different metrics, we reclassified as pathogenic the three variants previously reported as VUS, namely m.5645G > A/*MT-TA,* m.5865T > C*/MT-TY* and m.12283G > A*/MT-TL2,* contributing to consolidating the diagnostic algorithm. Except for one case, the predominant clinical presentation among patients was mitochondrial myopathy, characterized by COX– and RRFs on muscle biopsy, typically associated with PEO and ptosis, and frequently accompanied by sensorineural deafness. Retinal dystrophy was observed in three patients, while ataxia, dysphagia, peripheral neuropathy, gastrointestinal dysmotility, and parkinsonism consistent with Parkinson’s disease (PD) were each reported in single cases. The occurrence of PD is of particular interest—when attributable to mitochondrial dysfunction—as it is typically associated with nuclear genetic defects causing secondary mtDNA deletions or depletion, and only rarely with primary mtDNA point mutations.^35,36^ A coincidental association cannot be excluded, given the prevalence of PD in the general population, however nuclear variants linked to monogenic PD or common genetic risk factors (e.g. *GBA* variants) were not identified in this patient (data not shown). Notably, a single patient carrying the m.12145T > C variant in tRNA^His^ did not exhibit PEO/ptosis but instead presented with stroke-like episodes, consistent with a MELAS phenotype. This clinical manifestation is compatible with mt-tRNA mutations, and previous reports have documented MELAS associated with tRNA^His^ variants.^38^ Another case that somehow had a peculiar phenotype is Patient H, who presents a cachectic habitus possibly due to severe dysphagia and gastrointestinal dysmotility, while skeletal muscle displayed a bulk low heteroplasmic load of the pathogenic variant. We deepened our investigation of this case in the attempt to reconcile the low heteroplasmic load in skeletal muscle. Re-examination of the WES highlighted a heterozygous variant in the *SUCLA2* gene classified as VUS but never observed in homozygosity in GnomAD 4.1. SUCLA2 is known for affecting mitochondrial maintenance and the only feature to comment in our patient was that she did not display the usual compensatory activation of mitochondrial biogenesis, assessed as mtDNA copy number in skeletal muscle.^37^ We hypothesize that the *SUCLA2* variant may have somehow hampered mitobiogenesis, contributing the phenotype despite the low heteroplasmic load of the putative tRNA pathogenic variant. Overall, the clinical spectrum observed in this cohort is generally consistent with that reported for mtDNA point mutations affecting mt-tRNA genes.^16-24^

The case of Patient E, with two variants, also deserves further comments. Our analysis, taking advantage of the long reads sequencing, documented the occurrence of all four possible mitogenome combinations—wild type mtDNA, mitogenomes carrying both variants in *cis* and mitogenomes carrying each individual variant. This condition of tetraplasmy indicates the highly probable occurrence of mtDNA recombination, which is well documented but still unclear regarding the exact mechanisms and frequency of occurrence in different human tissues.^38,39^ Beyond recombination, the presence of double variants raises the critical question of which mutation primarily contributes to the clinical phenotype. In our case, despite pathogenicity predictions, only the m.10009G > A variant showed evidence of negative selection in blood, a feature commonly associated with severe mutations such as the canonical MELAS variant m.3243G > A.^40,41^ In our case, the m.10009G > A variant is also absent in the maternal relatives whereas the m.15961G > A variant is segregated in one proband’s daughter, who remains currently unaffected. Furthermore, in the dissected single muscle fibres the COX– fibres displayed high, parallel heteroplasmic load of both variants, and similarly both were under threshold with lower heteroplasmy in the COX+ fibres. Interestingly, the m.15961G > A variant is present in gnomAD 3.1 in 3 instances, 2 homoplasmic and 1 heteroplasmic, whereas the m.10009G > A variant is not reported. Thus, ultimately both variants might contribute to the phenotype of the proband, with the m.15961G > A variant being most likely equivalent to a hypomorphic variant and only by dissecting individually the two variants in the cybrid system we might discriminate the real weight of each variant, and their possible synergic interaction.

In our case series, we have been able to segregate the variants only in a few other maternal relatives. In Patient A, the variant was absent in blood cells and urinary sediment from four proband sisters, and in 1 nephew. This finding suggests a possible *de novo* event in the proband. In Patient B, instead, low levels of mutant heteroplasmy were documented in urinary sediment from the proband’s mother (7%) and brother (2%), demonstrating the presence of this pathogenic variant in the germline, being maternally inherited. Finally, for Patients C and H, the only other relatives investigated, respectively the sister and the brother, were negative in urinary sediment and blood cells. Therefore, no clear hypotheses can be formulated in the last two cases, due to the unavailability of further relatives and samples. Overall, in Patients D, F, G and I segregation analysis was not possible due to unavailability of relatives’ samples.

The single muscle fibre analysis provided, as previously suggested, an important indication corroborating the pathogenic role of the variants found, as in all cases the COX– fibres had greater mutant heteroplasmic load as compared to COX+ fibres. However, based on the current findings, we also have evidence suggesting that the threshold at which these variants lead to COX deficiency and, thus, the clinical manifestation of the disease may vary among different mutations. For example, Patient F, who did not display PEO/ptosis but had stroke-like episodes, displayed 0.1% COX– fibres essentially homoplasmic mutant (99% heteroplasmy), whereas the COX+ fibres had a mean heteroplasmy of 77% mutant load, which is exceedingly higher than the other patients of this case series. Consequently, the bulk heteroplasmy was 76%, possibly suggesting that the threshold for COX deficiency in this mutation is very high. It remains unclear if this finding relates to the central nervous system phenotype of MELAS, as opposed to the generally prevalent myopathic phenotype of the other cases. On the opposite end of the spectrum, Patient H, who displayed 2% of COX– fibres with 97% variant heteroplasmy, had only 1% of heteroplasmic load for the variant in COX+ fibres.

Increase of mitochondrial biogenesis is a known compensatory rewiring orchestrated by cells in the presence of OXPHOS deficiency.^42^ Our single cell muscle fibre analysis was further useful to highlight this compensatory reaction as on average the COX– fibres had a clearcut activation of mitochondrial biogenesis as exemplified by the assessment of mtDNA copy number when compared with COX+ fibres in the same patient. However, some patients displayed better efficiency than others in orchestrating this compensatory rewiring. For example, Patient I had the strongest increase of mtDNA amount in COX– fibres (+400%), whereas Patients C and H showed the lower one. The haplogroup background has been implicated in modulation of mtDNA copy number, and Patient I had haplogroup K, which was previously reported to be particularly efficient in activation of mitochondrial biogenesis.^43^ Conversely, Patient H lower amount of mtDNA, as discussed, may result from nuclear genetic modifiers.

Our further analysis of mtDNA rearrangements, while confirming the absence of any single or multiple deletions at heteroplasmic loads compatible with a pathogenic role, revealed that the age-dependent accumulation in patients was significantly higher than in controls, possibly due to an earlier generation of deleted molecules. This observation, which may suggest an accelerated ageing of mitochondrial patients, deserves further investigations on larger cohorts.

In conclusion, we described four novel pathogenic variants and reclassified the pathogenicity of previously reported variants affecting mitochondrial tRNA genes in a series of patients with myopathic phenotypes as key clinical feature. Our systematic approach, particularly the use of single muscle fibre analysis, provided key adjunctive information on mitochondrial biology of these variants, reporting the occurrence of mtDNA recombination in one patient and activation of mitochondrial biogenesis in COX deficient muscle fibres. This kind of analysis greatly improves the understanding of variant pathogenicity, underscoring the importance of this validation tool for which the availability of muscle biopsy remains central.

## Supplementary Material

fcag178_Supplementary_Data

## Data Availability

The data that support the findings of this study are available from the corresponding author, upon reasonable request.
