# Supplementary material for: Expanding the landscape of tRNA pathogenic variants in mitochondrial DNA
Source: Brain Commun. 2026 May 20;8(3):fcag178. doi: 10.1093/braincomms/fcag178 (PMC13233312; doi:10.1093/braincomms/fcag178)
Supplement: fcag178_Supplementary_Data [file fcag178_supplementary_data.pdf]

Supplementary Material

Supplementary figure

Medical histories

Supplementary figure

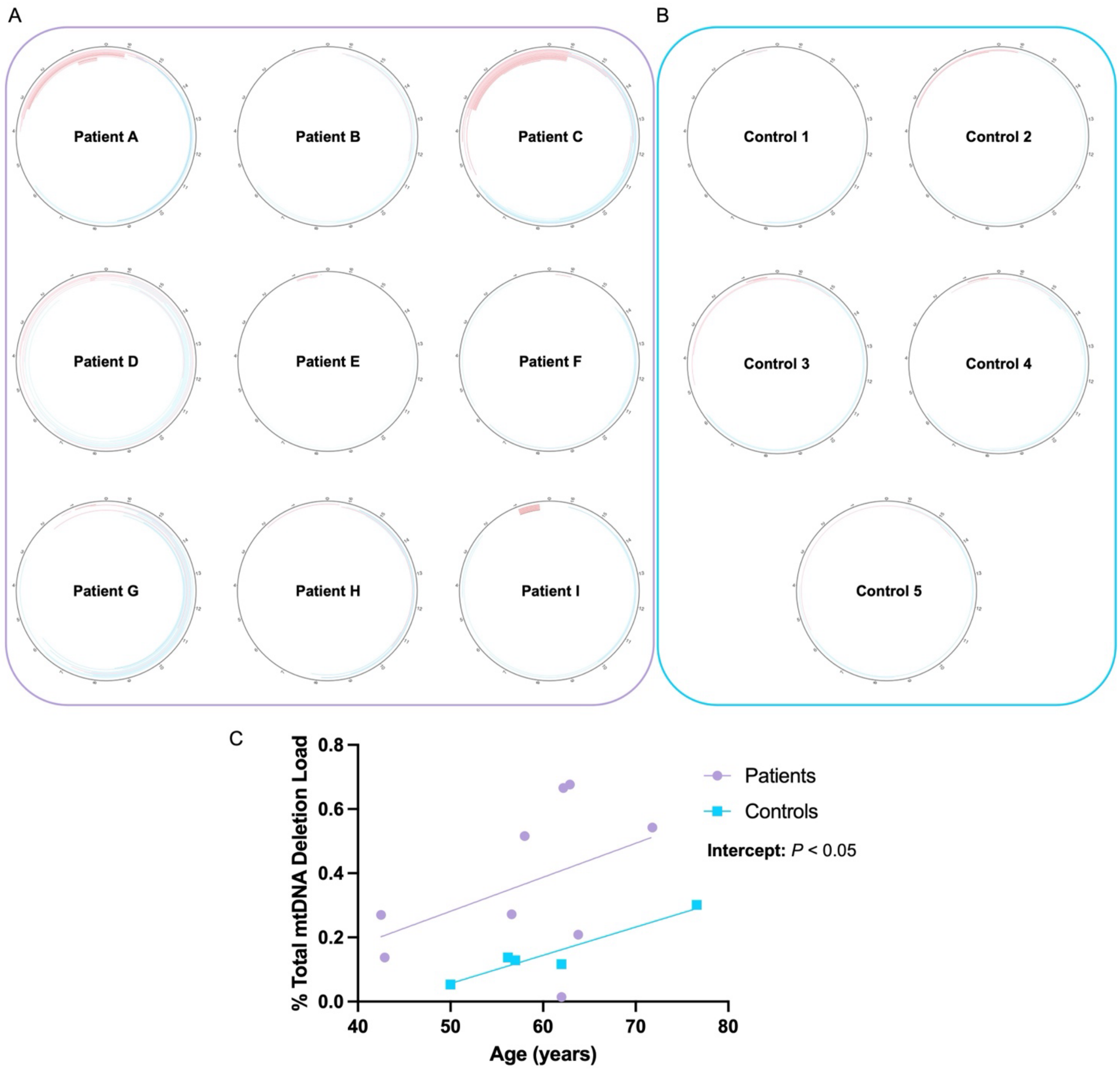

Supplementary Figure 1. Age-related accumulation of mitochondrial DNA structural rearrangements in patients and controls. Circular plots show mitochondrial DNA (mtDNA) structural rearrangements detected in skeletal muscle samples from patients (n = 9, panel **A**) and controls (n = 5, panel **B**). Each circle represents one individual mitogenome. mtDNA deletions are indicated in blue, whereas mtDNA duplications are indicated in red. Rearrangements are distributed along the mitochondrial genome circumference according to their genomic position, and their color intensity reflects relative abundance.

Simple linear regression analysis of total mtDNA deletion load as a function of age in patients (violet circles) and controls (cyan squares) (**C**). Comparison of regression slopes revealed no significant difference between patients and controls ($P = 0.8830$), indicating comparable age-dependent rates of mtDNA deletion accumulation in the two groups. In contrast, comparison of regression intercepts (elevations) showed a significant difference between groups ($P = 0.0399$), indicating a higher baseline mtDNA deletion load in patients compared with controls.

Medical histories

Patient A

This 76-year-old male first developed extrapyramidal signs at the age of 60. Bilateral ptosis had been noticed prior to the onset of extrapyramidal symptoms, although the exact time of onset was not documented. Neurological examination revealed bilateral ptosis, mild ophthalmoplegia, unilateral bradykinesia, and plastic rigidity, with no evidence of muscle weakness.

At 62 years of age, a DAT-SPECT demonstrated a bilateral, asymmetric reduction in dopamine transporter uptake in the putamen and caudate nuclei, consistent with impaired nigrostriatal dopaminergic transmission.

Given the presence of ptosis, a muscle biopsy was performed, which revealed mitochondrial myopathy with ragged-red fibers (RRF) and COX-negative fibers. Laboratory studies showed normal creatine kinase (CK) levels but elevated blood lactate after standardized exercise (36.4 mg/dl; normal range 5–22). Electromyography was normal. Audiometry documented mild sensorineural hearing loss. Cerebral MRI with proton spectroscopy revealed signs of chronic vascular encephalopathy, enlargement of the lateral ventricles, and increased cerebral lactate. The patient started levodopa therapy, which improved rigidity, and was supplemented with idebenone (405 mg/day), lipoic acid, creatine, and nicotinamide riboside.

Initial genetic testing of nuclear genes associated with mitochondrial myopathy and Parkinson's disease was negative. Full mtDNA sequencing from muscle subsequently identified the known pathogenic heteroplasmic variant m.3252A>G, with a heteroplasmy level of 37%.

From age 70 onwards, the patient experienced progressive worsening of Parkinson's disease, requiring escalation of dopaminergic therapy. At age 73, enteral administration of levodopa/carbidopa was initiated, resulting in improvement of motor symptoms.

Patient B

This 52-year-old male reported proximal muscle weakness since childhood (onset at age 7), characterized by difficulty running, climbing stairs, and rising from the ground. At age 9, scoliosis and elevated CK levels were documented, and a muscle biopsy led to a diagnosis of congenital myopathy. Right ptosis developed at age 10, becoming bilateral by age 13 and associated with ophthalmoplegia. Symptoms progressed slowly over time, and at 52 years he was also diagnosed with diabetes.

At 30 years of age, he developed progressive visual loss in the left eye and bilateral hearing loss. Family history was negative. First evaluation in our clinic occurred at 43 years of age, when neurological examination revealed bilateral ptosis with ophthalmoplegia, reduced visual acuity in the left eye, dysarthria, diffuse muscle atrophy, and pectus excavatum. Proximal muscle weakness was present in both upper and lower limbs (Medical Research Council [MRC] grade 4/5). Additional findings included reduced vibration sense (hypopallesthesia) and an ataxic gait.

Laboratory investigations showed elevated CK (1800 U/L; normal <170), lactic acidosis after standardized aerobic effort (83.4 mg/dl; normal 5–22), and a slight increase in glycated hemoglobin (43 mmol/mol; normal 20–42). Electromyography revealed myopathic changes with mild sensory polyneuropathy. Audiometry documented severe pantonal hypoacusis. Spirometry showed moderate restrictive insufficiency, without the need for ventilatory support. Cerebral MRI revealed cerebral and cerebellar atrophy. Neuropsychological testing indicated mild cognitive impairment, and

ophthalmological evaluation confirmed visual impairment, more marked in the left eye (visual acuity: right 0.8; left 0.2), due to retinal dystrophy.

A second muscle biopsy revealed mitochondrial myopathy with ragged-red fibers and COX-negative fibers. mtDNA sequencing identified the heteroplasmic variant m.3279C>T, with a heteroplasmy level of 34.7%.

The patient started supplementation with ubidecarenone (200 mg/day), lipoic acid, and carnitine.

Patient C

A 75-year-old female presented with mild ptosis beginning at age 60, which remained stable over subsequent years. She came to clinical attention due to disabling lower-limb pain and paresthesia.

Neurological examination revealed mild bilateral ptosis with ophthalmoplegia and mild proximal weakness of the lower limbs (MRC grade 4/5). Laboratory studies showed normal CK levels. Electromyography demonstrated focal myopathic changes, while lumbosacral MRI revealed vertebral canal stenosis. A muscle biopsy confirmed mitochondrial myopathy with COX-negative fibers. Full mtDNA sequencing from muscle identified the heteroplasmic variant m.5645G>A in MT-TA (45%).

Patient D

In this 79-year-old male, bilateral ptosis had slowly progressed since after age 40. He was initially diagnosed with ocular myasthenia and treated with pyridostigmine, without clinical benefit.

By age 62, neurological evaluation showed severe bilateral ptosis with mild ophthalmoplegia, without skeletal muscle weakness or fatigability. Laboratory tests demonstrated normal CK and basal lactate, with a mild post-exercise increase (30 mg/dl; normal 5–22). Electromyography was normal, while audiometry documented sensorineural hearing loss. Muscle biopsy showed mitochondrial myopathy with several COX-negative fibers.

Testing for mtDNA single deletions was negative. Full mtDNA sequencing from muscle identified the heteroplasmic variant m.5865T>C in MT-TY (43%). Treatment with idebenone (405 mg/day) was initiated at diagnosis.

Over 17 years of neurological follow-up, the patient showed progressive worsening of ptosis and deafness, without additional clinical manifestations. Cardiological and respiratory evaluations remained unremarkable.

Patient E

Visual impairment due to retinitis pigmentosa had been present in this 75-year-old female since age 18, progressively worsening over time and leading to severe visual disability. At age 50 she developed slowly progressive bilateral ptosis.

At 62 years of age, neurological examination showed bilateral reduction in visual acuity, bilateral ptosis with mild ophthalmoplegia, and mild, symmetrical proximal weakness of both arms and legs (MRC grade 4/5). Laboratory studies revealed normal CK but elevated blood lactate after standardized exercise (58.2 mg/dl; normal 5–22). Electromyography demonstrated myopathic changes. Cardiological and respiratory examinations were unremarkable. Brain MRI showed mild vascular encephalopathy and cortical atrophy. Muscle biopsy revealed mitochondrial myopathy with numerous ragged-red and COX-negative fibers. Full mtDNA sequencing identified two heteroplasmic variants, m.10009G>A in MT-TG and m.15961G>A in MT-TP, with heteroplasmy levels of 61.4% and 70%, respectively.

During follow-up, the patient showed progressive worsening of ptosis and visual impairment without new neurological manifestations. Treatment with idebenone (405 mg/day) was initiated at diagnosis.

Patient F

This 56-year-old male was admitted for subacute onset of involuntary movements of the shoulder and lower limbs. Brain MRI revealed a cortico-subcortical signal abnormality in the left temporo-occipital lobe without diffusion restriction, suggestive of a stroke-like episode; MR spectroscopy demonstrated a lactate peak and reduced N-acetyl aspartate. EEG showed biphasic slow spikes at 1 Hz with left predominance, and visual field testing confirmed right lateral homonymous hemianopsia.

Past medical history included bilateral hearing loss, headache, chronic diarrhea, and multiple episodes of intestinal obstruction, leading to clinical suspicion of MELAS syndrome. Muscle biopsy demonstrated few COX-negative fibers.

Genetic analysis excluded the common m.3243A>G/MT-TL1 MELAS mutation, but full mtDNA sequencing identified the heteroplasmic variant m.12145T>C/MT-TH (75.8%). Segregation studies were not performed due to loss to follow-up.

Patient G

This 67-year-old female developed bilateral ptosis with ophthalmoplegia at age 20, which progressed slowly and required surgical correction at age 58. At age 40, she reported exercise intolerance and mild proximal weakness of both arms and legs (MRC 4/5), which remained stable. Audiometry at age 60 documented mild hearing loss. Initially misdiagnosed with myasthenia, she was treated with pyridostigmine for many years without benefit.

At age 62, muscle biopsy revealed mitochondrial myopathy with ragged-red fibers and COX-negative fibers. Full mtDNA sequencing identified the heteroplasmic variant m.12283G>A/MT-TL2 (39.5%). Pyridostigmine was discontinued, and she was started on supplementation with ubidecarenone (200 mg/day) and creatine (200 mg/day).

Patient H

This 65-year-old female developed slowly progressive bilateral ptosis from age 25, followed five years later by surgical treatment for bilateral cataract and left ptosis. She subsequently developed ophthalmoplegia and progressive proximal and axial weakness, especially involving cervical musculature. From age 52 she experienced severe gastrointestinal dysmotility, including pseudo-obstruction, and by age 59 required parenteral nutrition. At age 63, cognitive decline emerged, confirmed by neuropsychological testing (MMSEc: 24). CSF analysis showed increased neurofilaments, tau, and phospho-tau, with a reduced beta-amyloid 1-42/1-40 ratio.

Additional investigations revealed bilateral deafness (audiometry), severe respiratory insufficiency with diaphragmatic paralysis (spirometry), bilateral macular dystrophy (ophthalmology), and mild cerebral atrophy without lactate accumulation (MRI spectroscopy). Two muscle biopsies (ages 40 and 58) consistently showed ragged-red fibers and COX-negative fibers. Nuclear gene testing for mitochondrial disorders was negative. Full mtDNA sequencing identified the heteroplasmic variant m.12315G>A/MT-TL2 (8.6%).

At her last examination (age 65), she presented cachexia, severe cervical weakness (MRC 1/5–2/5), bilateral ptosis with ophthalmoplegia, weakness of facial and masticatory muscles, hypophonia, and diffuse proximal weakness (MRC 2/5). She remained ambulant with ataxic gait and needed support. Supportive therapy included ubidecarenone (300 mg/day), riboflavin (200 mg/day), and nicotinamide riboside (750 mg/day).

Patient I

This 49-year-old male presented with unilateral ptosis and bilateral ophthalmoplegia at age 33, with very slow progression. He did not report skeletal muscle weakness or fatigability, and family history was unremarkable.

Laboratory tests showed elevated CK (1000 U/L). At age 43, muscle biopsy revealed mitochondrial myopathy with COX-negative fibers. Screening for mtDNA deletions was negative. Full mtDNA sequencing identified the heteroplasmic variant m.15923A>G/MT-TT (23.2%).
